# Design and Implementation of a Binary Phase-Shift Keying Frequency Diverse Array: Considerations and Challenges

**DOI:** 10.3390/s25010193

**Published:** 2025-01-01

**Authors:** Nicholas R. Munson, Bill Correll, Justin K. A. Henry, Ram M. Narayanan, Travis D. Bufler

**Affiliations:** 1 Department of Electrical Engineering, The Pennsylvania State University, University Park, PA 16802, USA; nrm5236@psu.edu (N.R.M.); jkh25@psu.edu (J.K.A.H.); 2 Maxar Intelligence, 1200 Joe Hall Drive, Ypsilanti, MI 48197, USA; bcorrell@ieee.org; 3 Applied Research Laboratory, The Pennsylvania State University, State College, PA 16801, USA; tdb15@arl.psu.edu

**Keywords:** frequency diverse array (FDA), binary phase-shift keying (BPSK), secure directional communications (SDCs), Universal Software Radio Peripheral (USRP), physical layer security

## Abstract

The frequency diverse array (FDA) is an architecture capable of beamforming in both range and angle, improving upon the traditional phased array (PA) which can only achieve beamforming in angle. The FDA employing directional modulation (DM) for secure directional communications (SDC) can reduce bit error rates (BERs) in both range and angle, again improving upon the traditional PA which can only reduce BER in angle. In this paper, we document the challenges involved in the design and implementation of a two-element linear FDA employing fast-time binary phase-shift keying (BPSK) modulations. We also show that the experimentally collected field data match well with the results of simulations based on our analytical model.

## 1. Introduction

The frequency diverse array (FDA) was first introduced in a series of works by Antonik in [1,2,3] and applied linear frequency offsets between uniformly spaced elements, generating range–angle coupled beampatterns with periodic modulations in range, angle, and time [4,5]. The resulting auto-scanning behavior is accomplished without the use of any other electronics or external phase-shifting hardware. Multi-mission, multi-mode applications such as simultaneous synthetic aperture and moving target indication have been proposed in [1,2,3]. Additional potential applications include volumetric scanning, beacons, and low-cost perimeter surveillance [6,7].

In contrast to the traditional phased arrays (PAs) that operate at a single carrier frequency, the FDA proposed in [1,2,3] radiates sinusoids, having small linear frequency offsets at each element of the array, creating a range dependence in the far-field pattern. The added pattern dependence on range allows for beamforming in both range and angle, improving upon the PA which can only perform beamforming in angle. Directional modulation (DM) techniques implemented on FDAs allow for bit error rate (BER) shaping in the range and angle dimensions [8,9,10,11,12], while DM using the PA can only reduce BER in angle [13,14]. The FDA also addresses the high costs and complexity of PA systems [6,7].

Although the ‘S’ shaped far-field patterns (see Figure 3a from [15]) produced by arrays with linear frequency offsets and uniform element spacing can eliminate clutter, far-field range periodicities result in range ambiguities [16]. By decoupling the range and angle dimensions, steerable localizations in energy can be created in the far-field pattern. Work in this area includes random FDA architectures [17,18], FDA sub-array architectures [19,20,21], non-linear frequency offsets [6,7,22,23,24,25,26], algorithmic optimizations of the FDA beampattern [27,28,29,30] and FDA wireless power transfer architectures [31,32,33].

In the literature, DM for FDAs has been well explored in quantitative studies [8,9,10,11,12], and techniques exist using fast-time coefficients to produce multiple point-like energy localizations in the FDA far-field pattern [27]. Although much theoretical work has been conducted, there exist no hardware implementations or builds (to the knowledge of the authors) in the area of FDA communications in the open literature, with the known array builds focused on radar functionality and pattern measurements. The current list of builds known to the authors includes the following:

- Antonik [34] (3×5 planar array using a 3×1 sub-array structure with five distinct frequencies);
- Brady [35] (two-element FDA waveform testbed);
- Eker [36] (1×10 linear frequency-modulated (LFM) FDA);
- Çetintepe [37] (re-built and improved the 1×10 LFM FDA in [36]);
- McCormick and Kellerman [38,39] (MISO LFM FDA with a 1×8 transmit array);
- Our previous work [40] (1×3 linear FDA using Ettus Universal Software Radio Peripheral (USRP) X310 software defined radios (SDR)).

The builds in [34,35,36,37,38,39,40] focus on radar implementations of the FDA. There also exists one hardware implementation by Huang [41,42] but with no array build.

In this paper, we continue the efforts in [34,35,36,37,38,39,40,41,42] and contribute to the experimental development of the FDA with a far-field test of a two-element linear FDA implementing binary phase-shift keying (BPSK) modulations on both elements with a 1MHz data rate. The goal of this work is to illustrate the steps needed to design, build, and test an FDA communication system with fast-time modulation in support of a future build and test of a DM FDA. The contributions of this paper are as follows:

1. We demonstrate the theoretical fast-time BPSK FDA signal model in practice, motivating further practical study of different FDA transmit–receive architectures.
2. We demonstrate the use of hardware employing commonly clocked digital-to-analog converters (DACs) to transmit frequency diverse signals having fast time-varying coefficients, motivating further practical study of FDA architectures employing structured fast-time modulation.

## 2. Directional Modulation Background and Practical Challenges

In a DM PA, phase shifts are applied at each element such that the complex summation of the far-field plane waves produces an altered constellation when sampled by a single channel receiver (see [13,14]). The alteration of the constellation, in turn, leads to the increased probability of bit errors. By appropriately designing the applied phase shifts, BERs can be controlled across the angle dimension.

In a DM FDA, it is common that the diverse radiated frequencies are processed on independent receive channels, and the complex summation of the synchronized channels achieves an altered constellation (see [9]). Increasing the number of elements yields more degrees of freedom when altering the constellation (i.e., more complex exponentials can be summed at the receiver, providing more control of the resultant amplitude and angle). The trade-off for the increased number of elements is an increase in the hardware channels required for the receiver.

The frequency diversity employed by the FDA allows for BER control in both range and angle, improving upon the PA which can only control BER in angle. It is clear that these approaches differ in concept and channel requirements at the receiver, incurring a trade-off with respect to the DM PA receiver simplicity and the DM FDA ability to control BER in range and angle. Alternative approaches to secure FDA DM exist in the literature. For example, the approach taken in [8] uses artificial noise sequences on transmit. However, we choose to start with the techniques described in [9] for the ease of practical implementation.

In [9], DM is modeled for an *N*-element linear FDA using quadrature phase-shift keying (QPSK). The signal model in Equation (5) in [9] sets up a vector-valued optimization of an additive length-*N* phase offset vector, wherein a genetic algorithm is used to iteratively refine the phase values for DM. The goal of such vector-valued optimization is that the coherent sum of the complex exponentials corresponding to the antenna elements should have very large amplitudes near the correct constellation points for desired range–angle locations and small amplitudes for incorrect constellation points for undesired range–angle locations. This idea is visually highlighted in Figure 8a,b in [9]. Here, we desire to undertake and address the practical considerations of building such a system.

The use of frequency diversity is key for range dimension beamforming; however, it also presents a practical challenge with respect to coherent, multi-frequency signal generation and reception. In this work, we test the multi-frequency phase synchronicity of a multi-frequency transmitter using a single channel SDR receiver to capture the radiated field behavior. The collected data allow us to test the analytical signal model and phase coherency of the transmit hardware. This test also serves as the first step in designing and actualizing a coherent multi-channel transmit–receive testbed required to implement the conceptual DM FDA architectures discussed in the academic literature.

In addition to testing the phase coherency of multi-frequency signal generation, we implement fast-time coefficients programmed on digital waveforms imported to a Keysight M8190A arbitrary waveform generator (AWG). The AWG is operated in uncoupled channel mode, allowing each transmit channel to operate independently while pulling from a common sample clock. To start sample streaming, the trigger condition is set as a positive-edge 0.5 V minimum waveform input. After the triggering event occurs, the samples on each respective channel are fed into their respective DACs and radiated into free-space via antennas. Sample synchronization is achieved via commonly clocked DACs. To generate a high-fidelity output signal from the AWG, we select an output frequency of 720 MHz, which is within the operational bandwidth given by the AWG baseband sample rate of 8 Gs/s.

## 3. Signal Model

**Figure 1:**
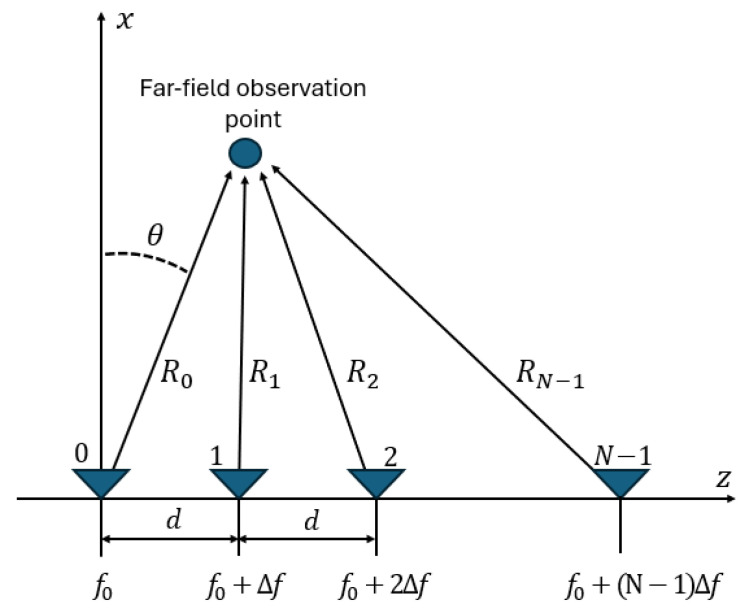
Schematic of a uniform linear FDA.

We begin with the signal model in [6,7] for a uniform linear FDA shown in Figure 1. The resultant far field for an array with *N* radiating elements having range $R_{n}=R-nd\sin\theta$ to the far-field point (R,θ) is given as

(1) $$F\left(t;R,\theta \right)=\sum_{n=0}^{N-1}e^{j2\pi f_{n}\left(t-R_{n}/c\right)}.$$

We include a general time-varying BPSK modulation parameter at the *n*th element $B_{n}\left(t\right)\in \left\{-1,1\right\}$, yielding

(2) $$F_{B_{n}}\left(t;R,\theta \right)=\sum_{n=0}^{N-1}B_{n}\left(t\right)\;e^{j2\pi f_{n}\left(t-R_{n}/c\right)}.$$

Since $B_{n}\left(t\right)$ is common to both terms in our modulation scheme, we can extract it outside the summation and let $B_{n}\left(t\right)\to B\left(t\right)$, yielding the new expression

(3) $$F_{B}\left(t;R,\theta \right)=B\left(t\right)\sum_{n=0}^{N-1}\;e^{j2\pi f_{n}\left(t-R_{n}/c\right)}.$$

## 4. BPSK FDA System Design

### 4.1. Linear Array Length Selection

The number of elements of the FDA with linearly progressive frequency offsets is inversely related to the mainbeam width (see Figures 5 and 6 in [6] for a comparison), which relates to the coherency window of the baseband waveform in our single channel receiver. Thus, we model the down conversion process by bringing Equations (2) and (3) to baseband as follows:

(4) $$\begin{array}{cc} F_{bb}\left(t;R,\theta \right) & =\left[\sum_{n=0}^{N-1}e^{j2\pi f_{n}\left(t-R_{n}/c\right)}\right]\cdot e^{-j2\pi f_{0}t}\\ \; & =\sum_{n=0}^{N-1}e^{j2\pi n\Delta f(t-R_{n}/c)}\cdot e^{-j2\pi f_{0}R_{n}/c} \end{array}$$

and

(5) $$\begin{array}{cc} F_{bb,B}\left(t;R,\theta \right) & =B\left(t\right)\left[\sum_{n=0}^{N-1}e^{j2\pi f_{n}\left(t-R_{n}/c\right)}\right]\cdot e^{-j2\pi f_{0}t}\\ \; & =B\left(t\right)\sum_{n=0}^{N-1}e^{j2\pi n\Delta f(t-R_{n}/c)}\cdot e^{-j2\pi f_{0}R_{n}/c}. \end{array}$$

**Figure 2:**
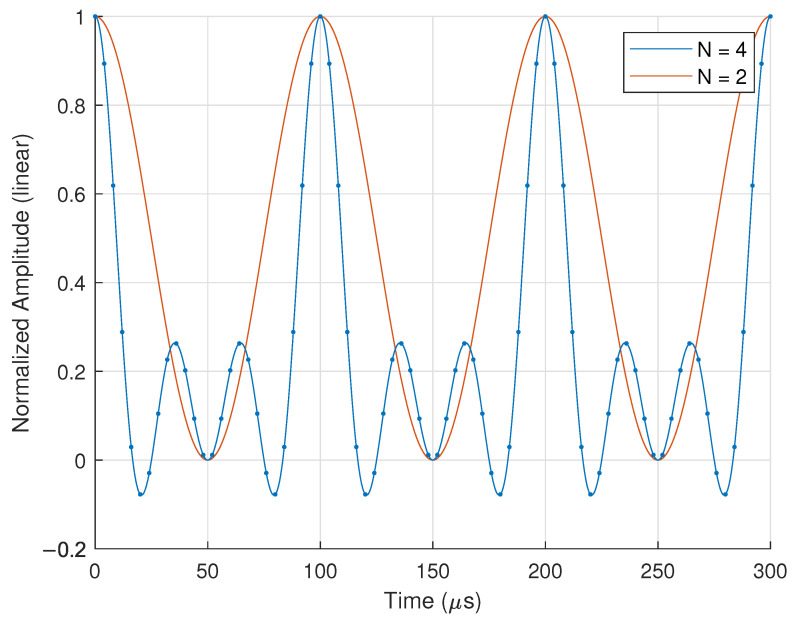
Simulated baseband behavior at a nominal far-field location (R,θ)=(10m,0°) for N=2 and N=4 element FDA with $f_{0}=720$ MHz and Δf=10 kHz.

We then plot the real component of Equation (4) with N=2 and N=4 elements, where $f_{0}=720$ MHz and Δf=10 kHz in Figure 2 at a nominal point in the far-field (R,θ)=(10m,0°).

By comparing the waveforms in Figure 2, we notice that the baseband waveform from the N=2 element FDA has a much wider mainbeam temporal window within which data can be modulated, as well as a simpler baseband behavior, and the N=4 element FDA produces a narrower beam with a tighter coherency window. This highlights a design trade-off between the mainbeam coherency window and mainbeam width with increasing linear FDA size.

Due to host PC limitations, we set our BPSK chip rate to $T_{c}=1\;\mu$s, yielding 10 samples per chip for the receiver with a receive sample rate of $f_{s}=10\;\mathrm{MHz}$. Thus, we select the N=2 array length for the longer coherency window, which allows for more bits per transmission pulse.

### 4.2. Baseband FDA Far-Field Behavior

Traditionally, a BPSK modulated signal has the general form [43]

(6) $$x\left(t\right)=A\left(t\right)\cos\left(2\pi f_{0}t\right),$$

where A(t)∈{−1,1} is the sign of the BPSK fast-time coefficient at fast time *t*, and $f_{0}$ is the carrier frequency.

In our BPSK FDA system with a single down-conversion frequency $f_{0}$ at the receiver, the baseband signal depends on the coherent summation of all radiated frequencies at the far-field point (R,θ). Thus, we evaluate Equation (4) with N=2

(7) $$\begin{array}{cc} F_{bb}\left(t;R,\theta \right)= & \;\;e^{-j2\pi f_{0}R/c}\;\;+\\ \; & e^{j2\pi \left(\Delta ft-\left(f_{0}+\Delta f\right)\frac{R}{c}+\left(f_{0}+\Delta f\right)\frac{d\sin\theta }{c}\right)}, \end{array}$$

then take the real component of the signal

(8) $$\begin{array}{cc} \; & F_{re,bb}\left(t;R,\theta \right)=\;\;\cos\left(2\pi f_{0}R/c\right)\;\;+\\ \; & \;\;\;\cos\left(2\pi \left[\Delta ft-\left(f_{0}+\Delta f\right)\frac{R}{c}+\left(f_{0}+\Delta f\right)\frac{d\sin\theta }{c}\right]\right). \end{array}$$

Equation (8) can be expressed as a single cosine wave with a range-periodic amplitude offset ($R_{p}=\lambda_{0}$) and temporal periodicity at fixed far-field locations ($T_{p}=1/\Delta f$). There is also a temporal shift in the maxima of each period given by

(9) $$\begin{array}{cc} \phi_{shift} & =2\pi \left[\left(f_{0}+\Delta f\right)\left(\frac{R}{c}-\frac{d\sin\theta }{c}\right)\right]\;\mathrm{mod}\;2\pi \\ T_{shift} & =T_{p}\left(1-\frac{\phi_{shift}}{2\pi }\right) \end{array}.$$

**Figure 3:**
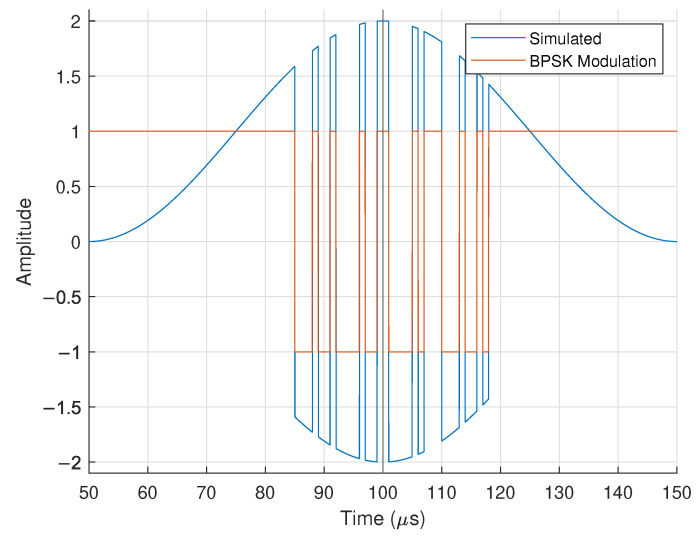
Simulated baseband response for a BPSK FDA with N=2, $f_{0}=720\;\mathrm{MHz}$, and Δf=10 kHz located at the far-field point (R,θ)=(2m,−25°).

These equations help determine how data should be modulated onto the BPSK carrier waves. For visualization, we show one period of a BPSK-modulated waveform in Figure 3, generated using Equation (5) with N=2, $f_{0}=720\;\mathrm{MHz}$ and Δf=10kHz at the far-field location (R,θ)=(2m,−25°). Note that we still assume perfect frequency and phase offset compensation at this point in the analysis.

**Figure 4:**
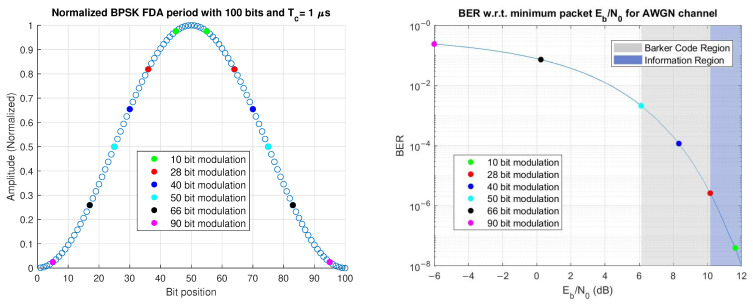
BER analysis to determine transmitted modulation size. The minimum amplitude of different packet lengths (**left**) are color-coded to match their respective points on the BER curve (**right**). The unit amplitude point (**left**) is assumed to have $E_{b}/N_{0}=12\;\mathrm{dB}$.

The design of this transmitted modulation window is based on the BER analysis shown in Figure 4. The bit at the maxima of the normalized amplitude is assumed (for now) to have an energy per bit ($E_{b}/N_{0}$) of 12dB. The energy per bit then scales with the squared amplitude of the waveform. The chosen modulation window is shown via the highlighted regions in Figure 4, where the BER with respect to minimum $E_{b}/N_{0}$ for varying modulation window sizes is shown. From Figure 4, it can be seen that the 50-bit window provides good balance between window size and BER.

### 4.3. Link Budget

When $E_{b}/N_{0}$ is increased, the overlaid scatter points in Figure 4 begin to shift clockwise on the BER curve. We desire that the Barker code region achieves a minimum BER of $10^{-5}$, requiring $E_{b}/N_{0}\approx 9.6\;\mathrm{dB}$. From [44],

(10) $$\begin{array}{cc} E_{b} & =\frac{P_{avg}}{R_{b}}\\ N_{0} & =\frac{P_{n}}{B_{n}}, \end{array}$$

where $P_{avg}$ is the average receive power, $R_{b}$ is the bit rate, $P_{n}$ is the noise power, and $B_{n}$ is the noise bandwidth. From [44,45],

(11) $$P_{n,dBm}=10\cdot \log_{10}\left(kTBF\cdot 1000\right),$$

where $k=1.38\cdot 10^{-23}\;J/K$ is Boltzmann’s constant, T=290K at room temperature and the noise factor $F=10^{NF/10}$. The noise figure (NF) of the TwinRX daughterboard with input frequency of 720MHz is at most NF=5 [46]. We use $B_{n}=2\;\mathrm{MHz}$ (the first null to first null bandwidth) instead of the sampling bandwidth B=10MHz as a buffer in the power calculation. Using Equations (10) and (11), the necessary receive power is found to be $P_{avg}=-89.1\mathrm{dBm}$.

The link between transmitter and receiver, in dB form, is, ref. [44]

(12) $$P_{r}=P_{t}+G_{t}+G_{r}-\mathrm{FSL},$$

where $P_{t}$ is the power transmitted, $G_{t}$ is the gain of the transmit antenna, $G_{r}$ is the gain of the receive antenna, and $\mathrm{FSL}={\left(4\pi R/\lambda_{0}\right)}^{2}$ is the free-space loss for the link. We measure $P_{t}=0\;\mathrm{dBm}$ at the transmit antenna terminals, and the log-periodic antenna gains are assumed to be $G_{t}=G_{r}=6.5\;\mathrm{dBi}$. For our close-range testing at R=2m, the power at the receiver is found to be $P_{r}=-31.5\;\mathrm{dBm}$, which is sufficient for the desired BER.

### 4.4. Channel Capacity

To ensure that appropriate channel capacity exists at our desired fast-time chip frequency, we evaluate the Shannon–Hartley theorem

(13) $$C=B\log_{2}\left(1+\frac{S}{N_{o}}\right)$$

with bandwidth B=10MHz and a signal-to-noise ratio of 7dB (determined analytically in Section 4.3), and find that $C=25.8\;\mathrm{Mb}/s\geq f_{chip}=1\;\mathrm{Mb}/s$. Note here that *S* and $N_{o}$ represent the signal level and noise level of the system, respectively.

### 4.5. Transmitted Packet Structure

The structure of one transmitted packet is shown in Table 1. Note the structure of Table 1 matches visually with the BPSK modulation in Figure 3 and Figure 4. The Barker code (BC) is used to bookend the bit information. The ‘buffer bits’ have a constant value of 1 and are used during regions without modulation.

### 4.6. Imported Transmit FDA Waveforms Discrete Model

The four parameters used to compute the size of the imported waveforms are as follows:

1. $T_{c}$: the desired length in time of a chip.
2. $N_{s,\;c}$: the number of samples per BPSK chip.
3. $N_{c}$: the total number of desired BPSK chips.
4. $N_{s}$: the total number of samples.

The discrete waveform model is

(14) $$s_{n}\left[m\right]=\sum_{i=0}^{N_{c}-1}b\left[i\right]\;\mathrm{rect}\left[m-iN_{s,c}\right]\cos\left[2\pi m/\left(f_{s}/f_{n}\right)\right],$$

where b[i] is the *i*th BPSK coefficient and

(15) $$\mathrm{rect}\left[m\right]=\left\{\begin{array}{c} \;\;\;1,\;\;\;\;\;\;m\in \{0,1,\cdots ,N_{s,c}-1\}\\ \;\;\;0,\;\;\;\;\;\;m\notin \{0,1,\cdots ,N_{s,c}-1\} \end{array}\right..$$

After computation, $s_{0}\left[m\right]$ and $s_{1}\left[m\right]$ are sent to Channels 1 and 2, respectively, of the M8190A AWG. A trigger is used to achieve a common start time between the channels, and coherency is retained through the use of commonly clocked ADCs.

We desire to use a discrete sinusoid with the ratio $f_{s}/f_{0}=10$ as our carrier wave. To fit within the operating bandwidth of the antennas and sample rate of the AWG, we set $f_{0}=720\;\mathrm{MHz}$, leading to a sampling frequency $f_{s}=7.2$ GHz. Table 2 provides a summary of the expected sample sizes required to implement Equation (14) when $T_{c}=1\;\mu$s and $N_{c}=100$. After computation, the waveform is exported from MATLAB R2020a and imported to the M8190A AWG.

## 5. System Outline

### 5.1. Transmit Array and Receive Probe Description

**Figure 5:**
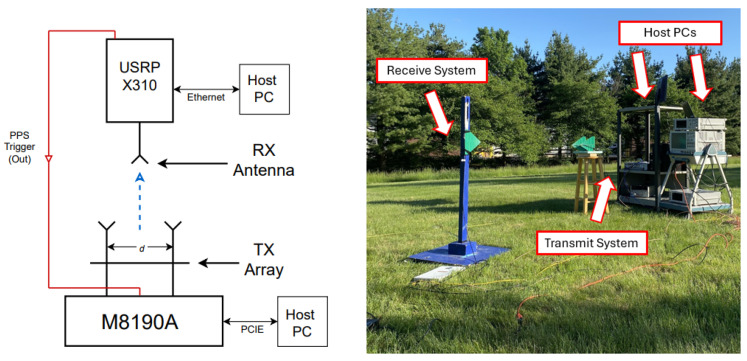
Block diagram (**left**) and photograph (**right**) of the BPSK FDA transmit–receive system. The blue arrow on the block diagram indicates the direction of wave propagation.

**Figure 6:**
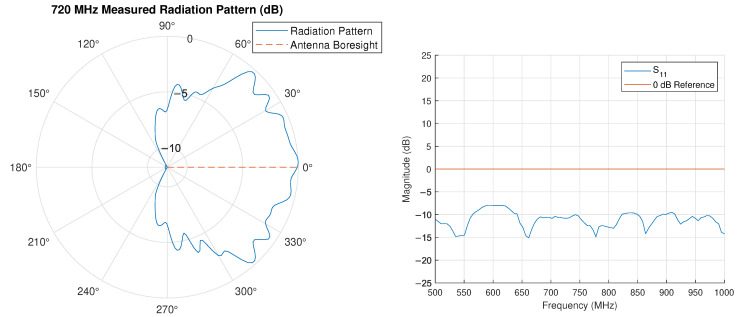
Measured antenna radiation pattern (**left**) and measured $S_{11}$ parameter (**right**).

The block diagram and photograph of the system are shown in Figure 5. The transmit array is a uniform linear array with N=2 elements, a designed carrier frequency $f_{0}=720$ MHz ($\lambda_{0}=c/f_{0}=0.416$ m, where *c* = 299,792,458 m/s is the speed of light), half-wavelength element spacings $d=\lambda_{0}/2=0.208$ m and far-field distance $R\geq 2D^{2}/\lambda_{0}=\lambda_{0}/2=0.104\;m$. The antennas used are identical ultra-wideband log-periodic antennas with operating range 600–6000 MHz. The measured antenna radiation pattern and measured $S_{11}$ parameter are shown in Figure 6. We make the monochromatic assumption in our far-field calculation because of the relative frequency band $\delta f=\frac{f_{max}-f_{min}}{f_{max}+f_{min}}=\frac{\left(f_{0}+\Delta f\right)-f_{0}}{\left(f_{0}+\Delta f\right)+f_{0}}\approx 0$ [47].

### 5.2. Transmit Hardware Description

The transmit hardware is an M8190A arbitrary waveform generator (AWG) (Keysight Technologies, Santa Rosa, CA, USA), which supports up to two channels per device and allows users to import custom waveforms created in MATLAB. The M8190A AWG has an 8 Gs/s sampling rate, and can achieve skew times of 6 ps between the two channels by adjusting the fine and coarse delays [48].

### 5.3. Receive Hardware Description

The receive hardware is a Universal Software Radio Peripheral (USRP) X310 (National Instruments, Austin, TX, USA) equipped with TwinRX daughterboards. The TwinRX supports 80 MHz of instantaneous bandwidth, and our host PC can stream captured baseband data at a maximum rate of 20 Ms/s.

## 6. Receiver Synchronization: Carrier Frequency Offset (CFO) Removal, Phase Rotation and Bitstream Generation

We assume the channel to be simply additive white Gaussian noise (AWGN), as our experiments are performed in an open field at high SNR. The AWGN channel model is

(16) s[n]=x[n]+w[n].

where s[·] is the received samples, x[·] is the transmitted signal, and w[·] is AWGN.

**Figure 7:**
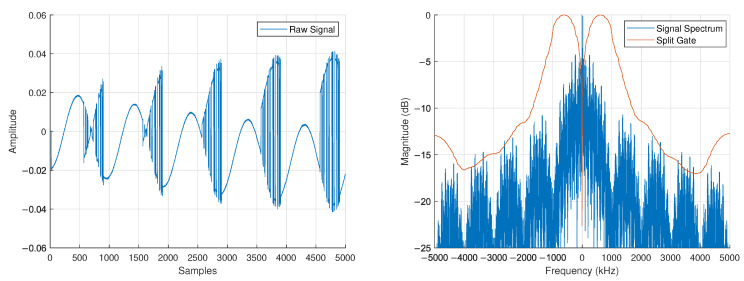
Captured baseband data before receiver synchronization (**left**) and associated spectrum (**right**) with a split gate used to find the approximate carrier location.

We assume the transmitting FDA will send short bursts of information in an SDC setting. Thus, we take in only $N_{rx}=5000\;\mathrm{IQ}\;\mathrm{samples}$ at the receiver at a rate of $f_{s}=10\;\mathrm{MHz}$, leading to a frequency resolution $f_{res}=1/t_{res}=f_{s}/N_{rx}=2\;\mathrm{kHz}$. We show the raw received signal in Figure 7. To acquire the carrier frequencies, we use a split-gate filter in the frequency domain. This filter is based on the fast-time chip length $T_{c}=1$ μs which yields a mainlobe bandwidth of $B_{ML}=2\frac{1}{T_{c}}=2\;\mathrm{MHz}$. The filter equation is given as

(17) $$H\left[n\right]=\left\{\begin{array}{cc} \;\;\;\;\frac{1}{H_{len}},\;\;\;\;\;\;\; & 0\leq n\leq \frac{t_{res}}{T_{c}}\\ -\frac{1}{H_{len}},\;\;\;\;\;\;\; & -\frac{t_{res}}{T_{c}}\leq n<0 \end{array}\right.,$$

where $H_{len}=2\frac{t_{res}}{T_{c}}$ is the length of the filter H[·].

The split-gate filter H[n] is correlated with the magnitude of the received signal |s[·]|:

(18) $$r\left[k\right]=\sum_{n}\left|s\left[n\right]\right|\cdot H\left[n+k\right].$$

The correlation r[k] is shown in Figure 7, where its minimum value provides the approximate center of the received spectrum and location of the carrier.

**Figure 8:**
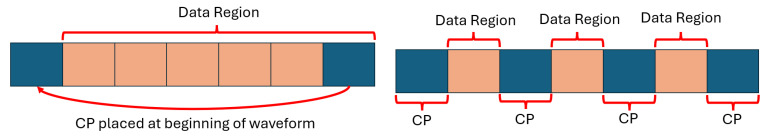
Similarities in the structure of an OFDM CP (**left**) and the BPSK FDA CP (**right**).

**Figure 9:**
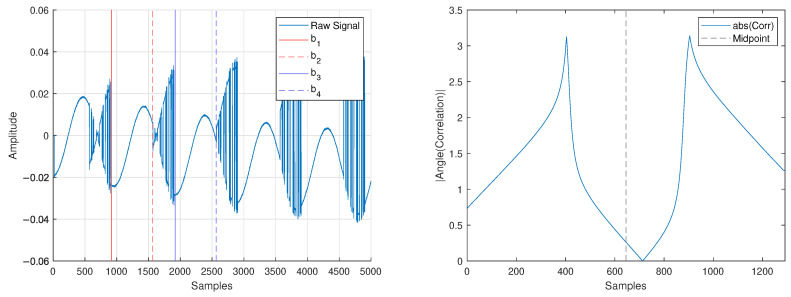
Captured baseband data before receiver synchronization (**left**) and phase information from the correlation of regions $b_{1}\leq n\leq b_{2}$ and $b_{3}\leq n\leq b_{4}$ (**right**).

In an orthogonal frequency division multiplexing (OFDM) communications signal, a portion of the signal at the end of the data transmission is placed at the beginning of the signal. This is called the cylic prefix (CP) and allows for a reference signal to compare against when trying to remove CFO. The parallel between OFDM CP and FDA CP is shown visually in Figure 8. In our BPSK FDA signal, the regions $b_{1}\leq n\leq b_{2}$ and $b_{3}\leq n\leq b_{4}$ (shown in Figure 9) provide the equivalent of a CP for the system.

After finding the approximate carrier location, we use the phase information from the auto-correlation of region $b_{1}\leq n\leq b_{2}$ and $b_{3}\leq n\leq b_{4}$ to identify and remove CFO present in Figure 7. The discrete-time baseband signal will have magnitude, phase, and CFO given by [49]

(19) $$s\left[n\right]=\left|s\left[n\right]\right|e^{j∠s[n]}\cdot e^{j2\pi n\Delta \nu /K},$$

where *K* is the number of samples in one temporal period $T_{p}$, and Δν is the CFO. The auto-correlation of s[n] and s[n+K] is

(20) $$\begin{array}{cc} s\left[n\right]\cdot s^{*}\left[n+K\right] & =|s\left[n\right]||s\left[n+K\right]|\;e^{j(∠s[n]-∠s[n+K])}\cdot e^{j2\pi \Delta \nu (n-(n+K))/K}\\ \; & ={\left|s\left[n\right]\right|}^{2}\cdot e^{j2\pi \Delta \nu } \end{array}$$

**Figure 10:**
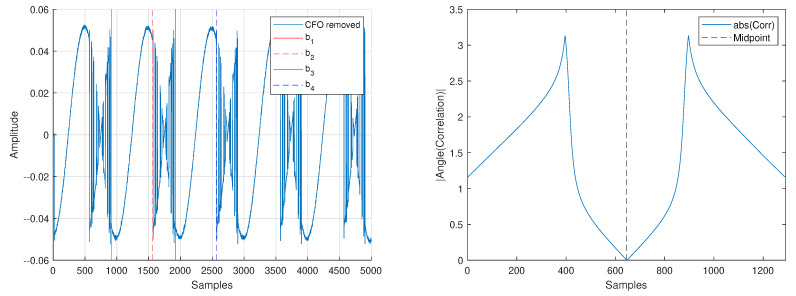
Captured baseband data with CFO removed (**left**) and phase information from the correlation of regions $b_{1}\leq n\leq b_{2}$ and $b_{3}\leq n\leq b_{4}$ (**right**).

Since |s[n]|=|s[n+K]| and ∠s[n]=∠s[n+K], the phase of the auto-correlation will allow us to remove CFO that exists in the received signal (see Figure 9 compared with Figure 10).

**Figure 11:**
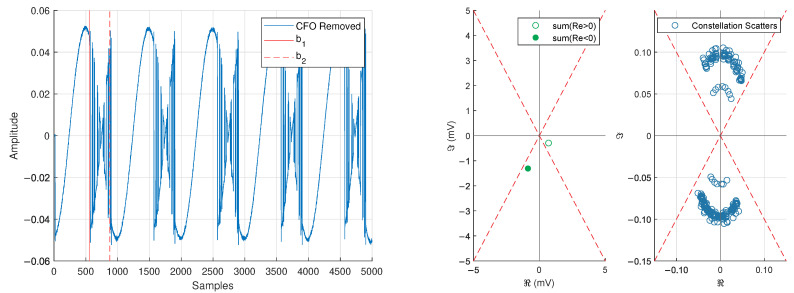
Captured baseband data with CFO removed (**left**) and constellation behavior of the modulation region $b_{1}\leq n\leq b_{2}$ (**right**).

We remove the phase offset with inspiration from a Costas loop. In our setup, we desire to keep the frequency offsets nΔf present in the baseband signal, and it is expected that the remaining carrier frequencies will cause a rotation of the constellation (see Figure 11). A traditional Costas loop will treat the additional carrier frequencies as an error signal and try to remove them [50,51]. Thus, we take inspiration from the structure of a Costas loop (but do not use the loop itself) to remove the phase offset still present in the signal.

We first form the region $b_{1}\leq n\leq b_{2}$ as shown in Figure 11. Within this region, we create two sums based on the sign of the real component of the signal

(21) S1=∑n=0Nrx−1s[n]·ejϕoff,Re(s[n])>0S2=∑n=0Nrx−1s[n]·ejϕoff,Re(s[n])<0

**Figure 12:**
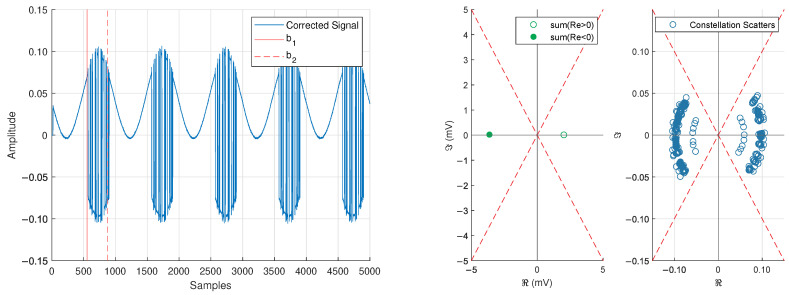
Captured baseband after receiver synchronization (**left**) and constellation behavior of the modulation region $b_{1}\leq n\leq b_{2}$ (**right**).

The desired compensation angle $\phi_{off}$ is such that $∠S_{1}$ and $∠S_{2}$ are driven as close to the desired values $∠S_{1}\to 0$ and $∠S_{2}\to \pi$ as possible. This output of this process is shown in Figure 12. Note that it is possible to lock ±π out of phase, but this can be corrected by checking the sign of a Barker code correlation.

To create the receive bitstream, we follow Figure 2 of [43]. The procedure is as follows:

1. Identify the bit transitions based on a zero-crossings vector.
2. Sum 10 samples of Re(s[·]), incrementing by 10 samples for each sum.
3. Choose 1 vs. 0 based on the sign (+1 for 1 and −1 for 0) of the summation.

## 7. Experimental Results

**Figure 13:**
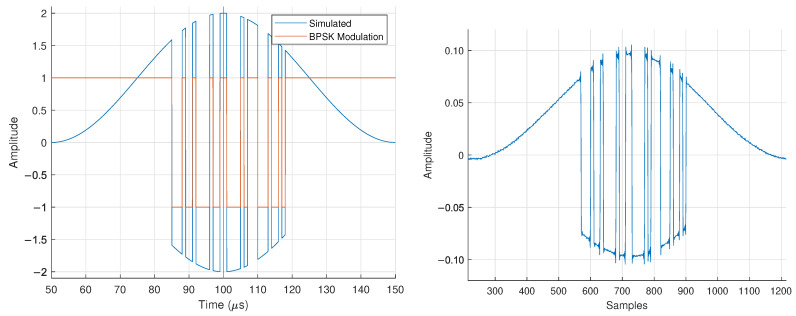
Simulated baseband BPSK FDA (**left**) and 100μs of the captured BPSK FDA data with receiver synchronization in Figure 12 (**right**).

We now show the full results of our experimental testing. In Figure 13, we show a 100μs zoom-in of the corrected signal shown in Figure 12, as well as the simulated baseband behavior. The signal has a temporal periodicity of 98.9μs that matches closely with the value expected from Equation (8) of $T_{p}=100\;\mu$s.

For continuity, we restate Equations (4) and (5) in their baseband form below:

(22) $$\begin{array}{cc} F_{bb}\left(t;R,\theta \right) & =\left[\sum_{n=0}^{N-1}e^{j2\pi f_{n}\left(t-R_{n}/c\right)}\right]\cdot e^{-j2\pi f_{0}t}\\ \; & =\sum_{n=0}^{N-1}e^{j2\pi n\Delta f(t-R_{n}/c)}\cdot e^{-j2\pi f_{0}R_{n}/c} \end{array}$$

and

(23) $$\begin{array}{cc} F_{bb,B}\left(t;R,\theta \right) & =B\left(t\right)\left[\sum_{n=0}^{N-1}e^{j2\pi f_{n}\left(t-R_{n}/c\right)}\right]\cdot e^{-j2\pi f_{0}t}\\ \; & =B\left(t\right)\sum_{n=0}^{N-1}e^{j2\pi n\Delta f(t-R_{n}/c)}\cdot e^{-j2\pi f_{0}R_{n}/c}. \end{array}$$

Taking the magnitude of Equations (22) and (23) yields

(24) $$\begin{array}{cc} |F_{bb}\left(t;R,\theta \right)| & =|\sum_{n=0}^{N-1}e^{j2\pi n\Delta f(t-R_{n}/c)}\cdot e^{-j2\pi f_{0}R_{n}/c}|,\\ |F_{bb,B}\left(t;R,\theta \right)| & =|B\left(t\right)\sum_{n=0}^{N-1}e^{j2\pi n\Delta f(t-R_{n}/c)}\cdot e^{-j2\pi f_{0}R_{n}/c}|. \end{array}$$

Using the identity $|z_{1}z_{2}\left|=\right|z_{1}\left|\right|z_{2}|$ (where $z_{1}$ and $z_{2}$ are complex numbers) and the fact that |B(t)|=1∀t, we see that $|F_{bb}\left(t;R,\theta \right)|$ and $|F_{bb,B}\left(t;R,\theta \right)|$ in Equation (24) should have the same fast-time behavior:

(25) $$\begin{array}{cc} |F_{bb}\left(t;R,\theta \right)| & =|\sum_{n=0}^{N-1}e^{j2\pi n\Delta f(t-R_{n}/c)}\cdot e^{-j2\pi f_{0}R_{n}/c}|\\ |F_{bb,B}\left(t;R,\theta \right)| & =|\sum_{n=0}^{N-1}e^{j2\pi n\Delta f(t-R_{n}/c)}\cdot e^{-j2\pi f_{0}R_{n}/c}|. \end{array}$$

**Figure 14:**
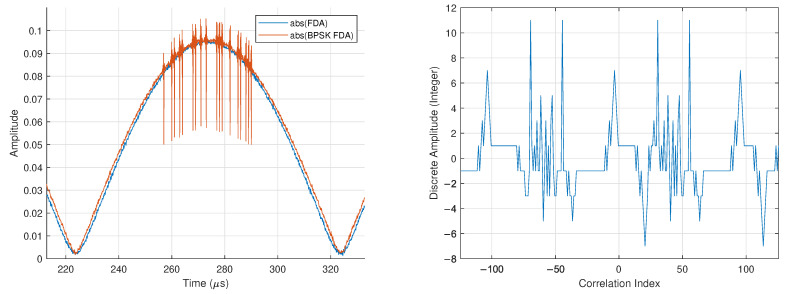
Experimental demonstration of Equations (24) and (25) (**left**) and length-11 Barker code correlation with 250 μs worth of bit information from the synchronized waveform in Figure 12 (**right**).

We show this in Figure 14, where the magnitude of an N=2 FDA with $f_{0}=720\;\mathrm{MHz}$ and Δf=10kHz is overlaid with the magnitude of the BPSK FDA. Also shown in Figure 14 is the correlation of 250 bits from the received bitstream with a length 11 Barker code. The correlation showcases periodic packets with bookended Barker codes, matching the expected behavior of the transmitted packet structure described in Table 1.

## 8. Summary, Future Work, and Discussion

This paper documents the build and test of an FDA communication system, which we hope to extend to a simultaneous FDA radar/communications system. In this work, a two-element linear FDA is used to transmit messages on the range–angle plane using BPSK modulations on both elements. The collected data enable us to evaluate the analytical signal model and phase synchronicity of the transmit hardware. This test represents the first step in designing and implementing a coherent multi-channel transmit–receive testbed, which is essential for realizing the conceptual DM-FDA architectures found in the academic literature. The theoretical baseband behavior of the received waveform when down-converted at the carrier frequency $f_{0}$ is characterized, then demonstrated via experimental results. The link between the transmitter and receiver is analyzed, and a transmit modulation scheme is developed with appropriate power considerations to achieve a desired BER. The modulated information is bookended using the length-11 Barker code, which marks both the beginning and ending of the encoded message. The received bitstream is validated with a discrete correlation of the received bitstream with the length-11 Barker code, as well as a cross-referencing of the received bitstream with the information bits programmed at the transmitter.

The DM FDA analysis presented in the academic literature often relies on a set of principles, which typically includes some (or all) of the following:

1. Phase-synchronous analysis that assumes the receiver is always frequency- and phase locked with the transmitter.
2. Neglecting spectrum spreading, with closely spaced spread spectrum signals that are assumed to be perfectly separable.
3. No use of training symbols. Orthogonal training symbols can be created via a time-division multiplexing approach. Thus, movement of the symbols on a constellation does not necessarily imply non-recoverability.

To address the points above, we generate a list of research questions that we will address in our future work:

1. What are the necessary requirements of the multi-channel coherent receive hardware?
2. What is the trade-off between the designed frequency offsets and tolerable spectrum spreading with respect to signal separability?
3. How can we measure the ‘zone-of-security’ generated by DM FDAs? Moreover, how can BER control be attributed to the DM FDA itself and not artifacts of practical analog signal conditioning, signal generation, and/or down conversion?

## Figures and Tables

**Table 1:** Packet structure with $N_{c}=100$. The **bold** entries are the modulated information.

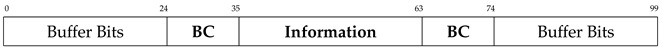

**Table 2:** Waveform sample size with $T_{c}=1\;\mu s$ and $N_{c}=100$.

Parameter	Expression	Samples
$N_{s,c}$	$T_{c}\cdot f_{s}$	7200
$N_{s}$	$N_{s,c}\cdot N_{c}$	720,000

## Data Availability

The datasets presented in this article are not readily available because of sponsor restrictions.
